# Is Migraine a Risk Factor for Non-Arteritic Anterior Ischemic Optic Neuropathy? Insights from a National Case–Control Study

**DOI:** 10.3390/brainsci16010082

**Published:** 2026-01-07

**Authors:** Itamar Ben Shitrit, Eyal Walter, Erel Domany, Nir Amitai, Tomer Kerman, Erez Tsumi, Assaf Kratz, Asaf Honig

**Affiliations:** 1 Joyce & Irving Goldman Medical School, Ben-Gurion University of the Negev, Be’er-Sheva 84101, Israel; 2 Clinical Research Center, Soroka University Medical Center, Be’er-Sheva 84516, Israel; 3 Department of Ophthalmology, Soroka University Medical Center, Be’er-Sheva 84101, Israel; 4 Faculty of Health Sciences, Ben-Gurion University of the Negev, Be’er-Sheva 84101, Israel; asaf.honig2@gmail.com; 5 Department of Neurology and Headache Clinic, Rambam Health Care Campus, Haifa 31096, Israel; 6 Department of Neurology, Brain Medicine Division, Soroka University Medical Center, Be’er-Sheva 84516, Israel

**Keywords:** non arteritic anterior-ischemic-optic-neuropathy, migraine, cerebral autoregulation, ophthalmic artery, congestive heart failure

## Abstract

**Purpose**: While migraine is linked to increased cerebrovascular risk, its association with non-arteritic anterior ischemic optic neuropathy (NAION) remains underexplored. **Methods**: We conducted a retrospective case–control study using population-based electronic medical records. NAION patients were compared to propensity score-matched controls regarding migraine prevalence and clinical characteristics. **Results**: From 2001 to 2022, among 6,566,619 patients, 1629 NAION cases (mean age 67 ± 13 years; 45% female) and 6433 propensity matched controls were identified. The prevalence of migraine was similar in both groups (3.8% vs. 3.3%, *p* = 0.3). Among migraine patients, those with NAION (n = 62, age 62 ± 11) and controls (n = 212, age 60 ± 11) had comparable baseline characteristics, except for congestive heart failure (9.7% vs. 2.4%, *p* = 0.027). Within the NAION cohort, migraineurs (n = 64) were younger (62 ± 12 vs. 67 ± 13 years, *p* < 0.001), and had lower rates of diabetes mellitus (35% vs. 57%, *p* < 0.001) and peripheral vascular disease (1.6% vs. 9.6%, *p* = 0.03). Female migraineurs developed NAION at a younger age than females without migraine (60 ± 12 vs. 69 ± 12 years, *p* < 0.001); no such difference was seen in males. Multinomial logistic regression revealed that migraine was independently associated with younger age at NAION onset, particularly in patients aged <59 (OR = 5.8, *p* = 0.001) compared with those >70. An independent 1:4 migraine to non-migraine matched cohort (n = 310) showed similar age-dependent trends. **Conclusions**: While migraine was not more prevalent among NAION patients, females with migraine developed NAION at a younger age and had fewer vascular comorbidities. Congestive heart failure was more prevalent among migraine patients who developed NAION, suggesting a potential contributory role of systemic hypoperfusion.

## 1. Introduction

Among the various optic neuropathies, non-arteritic anterior ischemic optic neuropathy (NAION) emerges as the leading cause of acute optic nerve injury in individuals beyond their fifth decade of life. Epidemiological data suggests an annual incidence of 10 per 100,000 individuals, with men showing slightly higher susceptibility [1,2]. Patients typically present with acute, painless vision loss, often noticed upon awakening [2,3]. The pathophysiology of NAION involves vascular insufficiency affecting the short posterior ciliary arteries, which leads to a cascade of ischemic events and optic nerve head edema. The confined space of the rigid scleral canal exacerbates this process, creating a compartment syndrome that perpetuates ischemia and ultimately results in irreversible damage to retinal ganglion cells and their axons [2,4]. Key risk factors for NAION include chronic cardiovascular conditions such as hypertension, diabetes, hyperlipidemia, and obstructive sleep apnea [1,4,5,6]. Additionally, an optic nerve head with a crowded appearance (referred to as a “disc at risk”) as well as nocturnal hypotension and anemia, which may induce hypoperfusion, are significant contributors to the condition [4,5].

Migraine, affecting over 1 billion people worldwide, is characterized by recurrent moderate-to-severe headaches, often with nausea, vomiting, photophobia, and phonophobia [7,8], with a female to male ratio of 3:1 [7,9]. Its prevalence peaks at ages 35–39 years and declines with age, dropping to around 7% at 60–69 years and 4% at 70 years or older [8,10]. This decline reflects a tendency toward remission in older age, particularly after 60 years, although the reasons for this remission remain poorly understood [11].

Migraine and cerebrovascular disorders have been linked through factors such as impaired cerebral autoregulation and cerebrovascular vasospasm [12,13]. Moreover, vasoconstrictive migraine treatments such as triptans may increase the risk of cerebrovascular events [14]. Review of the existing literature suggests that NAION may occur in patients with migraine at a younger age than is typical, with several shown cases reported in individuals under 50 years of age [15,16]. Studies suggest that the temporal association between migraine episodes and vision [17,18] loss could indicate vasospasm as a contributing factor to ischemic events at the optic nerve head, potentially playing a role in the development of NAION [17,18,19].

The association between migraine and NAION has been suggested in several case studies but has not been examined on a larger scale [15,16]. Here, we aim to investigate the age-related influence of migraine within a population of NAION patients.

## 2. Methods

### 2.1. Study Population

This retrospective case–control study utilized electronic medical records from Clalit Health Services (CHS), Israel’s largest health maintenance organization, covering approximately five million individuals (~51% of the national population). Data were collected between 1 January 2001, and 31 December 2022. CHS membership is stable, with an annual turnover of less than 1%, enabling reliable longitudinal analyses [20]. The study followed STROBE guidelines [21].

NAION cohort—Initially, we have identified NAION cases. This case group included patients diagnosed with NAION, as identified by the International Classification of Diseases (ICD-9) code 377.41. To ensure the validity of case identification and to exclude confounding conditions that might mimic NAION, individuals with a prior diagnosis of giant cell arteritis (ICD-9 code 446.5) or optic neuritis (ICD-9 code 377.30) were excluded from the cohort.

Propensity matched control cohort—For each NAION case, four controls matched by birth year and sex were selected. Propensity score matching adjusted for major systemic and vascular comorbidities, including diabetes, hypertension, peripheral vascular disease, cerebrovascular disease, congestive heart failure, renal disease, malignancy, and chronic pulmonary disease, based on ICD-9 codes (Table S1).

We identified migraine cases using ICD-9 codes 346.0–346.9, only when recorded before the diagnosis of NAION. We excluded individuals diagnosed with migraine after NAION to reduce the risk of including cases where a new headache and NAION could reflect undiagnosed temporal arteritis.

### 2.2. Data Sources and Variables

Anonymized patient data was extracted from the CHS comprehensive electronic medical record (EMR) system. This digital patient record system, documenting data since 2000, integrates real-time data from primary care, ambulatory services, and hospitals, ensuring high reliability, consistency, and accuracy of exposure and follow-up data. The data extraction was conducted using the CHS Data platform, which is powered by MD-Clone (https://www.mdclone.com) which ensures de-identification and privacy through advanced algorithms.

The dataset included demographic variables such as birth date, sex, socioeconomic status (three-level scale), and ethnicity (categorized as Jewish and Arab), alongside clinical variables representing chronic conditions and comorbidities. All diagnoses included in the dataset were recorded prior to the diagnosis of NAION.

### 2.3. Statistical Analysis

**Figure 1:**
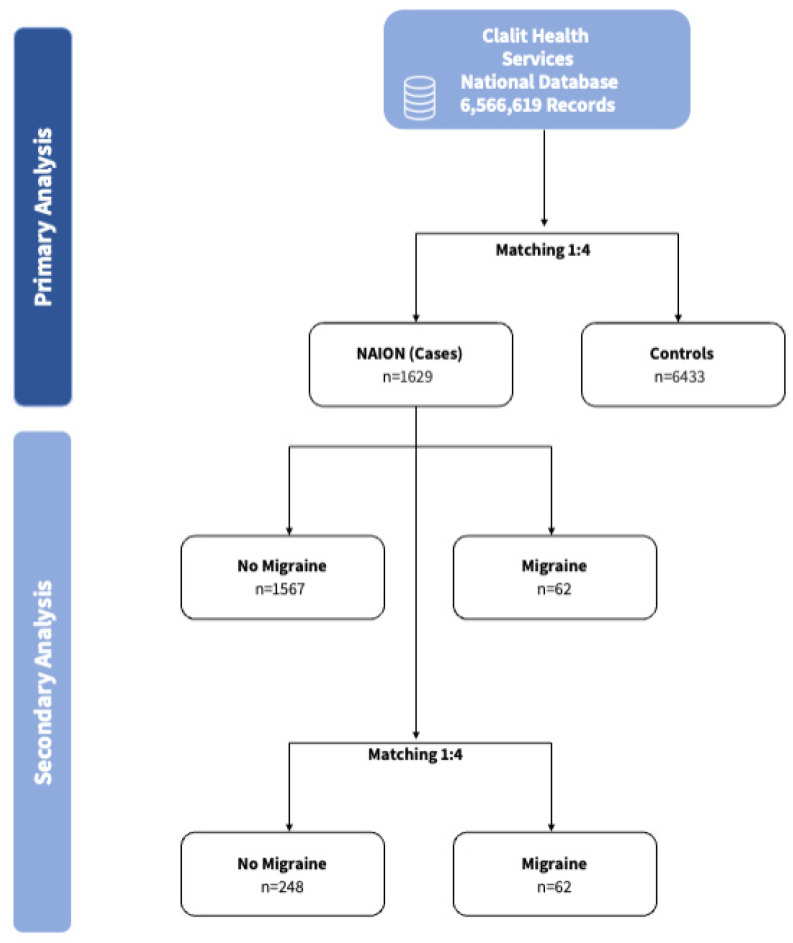
Flow diagram depicting the study selection process, including primary analysis with matched NAION cases and controls, and secondary analysis stratified by migraine status.

This study employed a preplanned three-step analytical approach (Figure 1). First, a case–control comparison between NAION cases and non-NAION propensity matched controls. Second, a subgroup analysis within the NAION population, comparing individuals with and without migraine, stratified by sex.

Continuous variables are presented as mean ± standard deviation and categorical variables as frequencies and percentages. Group comparisons employed parametric or non-parametric tests as appropriate. Multivariable multinomial logistic regression models were used to assess the association between migraine and age at NAION onset across predefined age strata, adjusting for established NAION risk factors. Propensity-score matching was used as a validation approach. Statistical significance was defined as a two-sided *p* value < 0.05.

To assess the interaction between age and migraine while reducing bias, we performed a propensity-matched analysis. NAION patients with and without migraine were matched at a 1:4 ratio using established NAION risk factors, ref. [4] as covariates, excluding age, aiming for a standardized mean difference of 0.1 or less.

All statistical analyses were performed using R version 4.4.0, with statistical significance defined as a two-sided *p* value less than 0.05.

### 2.4. Ethics Approval

The CHS ethical committee approved the study (approval number 0198-23-SOR) and granted a waiver of informed consent due to the retrospective nature of the research and the secondary use of anonymized clinical data.

## 3. Results

### 3.1. Comparison of NAION Cohort to Control Cohort

From 2000 to 2022, a review of 6,566,619 electronic patient charts identified 1629 cases of NAION (age of index event 67 ± 13, 45% females) for an incidence of 24.8 NAION cases per 100,000 individuals. A 4:1 propensity-matched control cohort was created with 6433 controls (Table S2). Comparable rates of migraine were found in the NAION and control cohorts (3.8% and 3.3%, respectively, *p* = 0.31). Similarly, in a sub-analysis of the female population, migraine rates were found comparable for the NAION and control cohorts (6.1% vs. 4.9%, respectively, *p* = 0.2). Although the populations were matched, we performed a multivariable logistic regression analysis using known predictors of NAION and migraine (Table S3). Hypertension (OR 1.69, *p* < 0.001) and peripheral vascular disease (OR 1.62, *p* = 0.018) emerged as independent predictors of NAION, whereas migraine was not an independent predictor (OR 1.17, 95% CI 0.87–1.57, *p* = 0.30). Moreover, even when performing a post hoc analysis limited to a population of females aged 65 years old and younger, migraine was not an independent predictor of NAION in a multivariate model (OR 1.64, 95% CI 0.72–3.76, *p* = 0.2).

To identify variables differentiating migraine patients who developed NAION from those who did not, we compared migraine patients in the NAION cohort (n = 62, age 62 ± 11 years) with migraine patients in the control cohort (n = 212, age 60 ± 11 years). Baseline characteristics were similar between groups, except for a higher prevalence of congestive heart failure (CHF) (9.7% vs. 2.4%, *p* = 0.027) (Table 1).

### 3.2. Analysis Within the NAION and Control Cohorts

Among NAION patients, those with migraine were more likely to be female (73% vs. 44%, *p* < 0.001), were diagnosed with NAION at a younger age (mean 62 ± 12 vs. 67 ± 13 years, *p* < 0.001), and have higher socioeconomic status (29% vs. 19%, *p* = 0.043). Notably, migraine patients had lower rates of any diabetes mellitus (35% vs. 57%, *p* < 0.001) or diabetes without complications (27% vs. 44%, *p* < 0.01) and PVD (1.6% vs. 9.6%, *p* = 0.03). Similarly, comparisons made among control cohort between patients with and without migraine (Table S4) found that migraine patients were younger (mean age 60 ± 11 vs. 68 ± 13, *p* < 0.001), were more likely to be females (68% vs. 44%, *p* < 0.001) and had lower rates of any diabetes mellitus (44.3% vs. 55%, *p* = 0.002).

**Figure 2:**
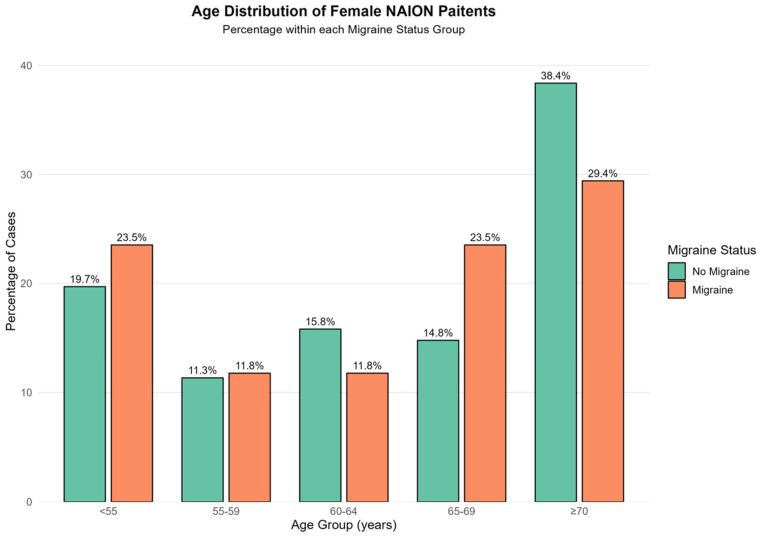
Age Distribution of female patients diagnosed with NAION by Migraine Status. This bar chart illustrates the percentage distribution of Non-Arteritic Anterior Ischemic Optic Neuropathy (NAION) cases across age groups (<55, 55–59, 60–64, 65–69, ≥70 years) stratified by migraine status. The green bars represent patients without migraine, while the orange bars represent patients with migraine. The percentages reflect the proportion of cases within each migraine status group.

Table 2 presents the background characteristics of NAION patients with and without migraine, stratified by sex. Among females, those with migraine were younger at the time of NAION diagnosis (60 ± 12 vs. 69 ± 12 years, *p* < 0.001). In contrast, among males, the age at NAION diagnosis was similar for patients with and without migraine (65 ± 13 vs. 65 ± 13 years, *p* = 0.58). Among female NAION patients younger than 55, migraine patients were more prevalent than non-migraine patients (23.5% vs. 19.7%, *p* < 0.001, Figure 2). Conversely, in the NAION female cohort aged 70 and older, non-migraine patients were more prevalent than migraine patients (38.4% vs. 29.4%, *p* < 0.001, Figure 2).

### 3.3. Multivariable Multinomial Logistic Regression Analysis

**Figure 3:**
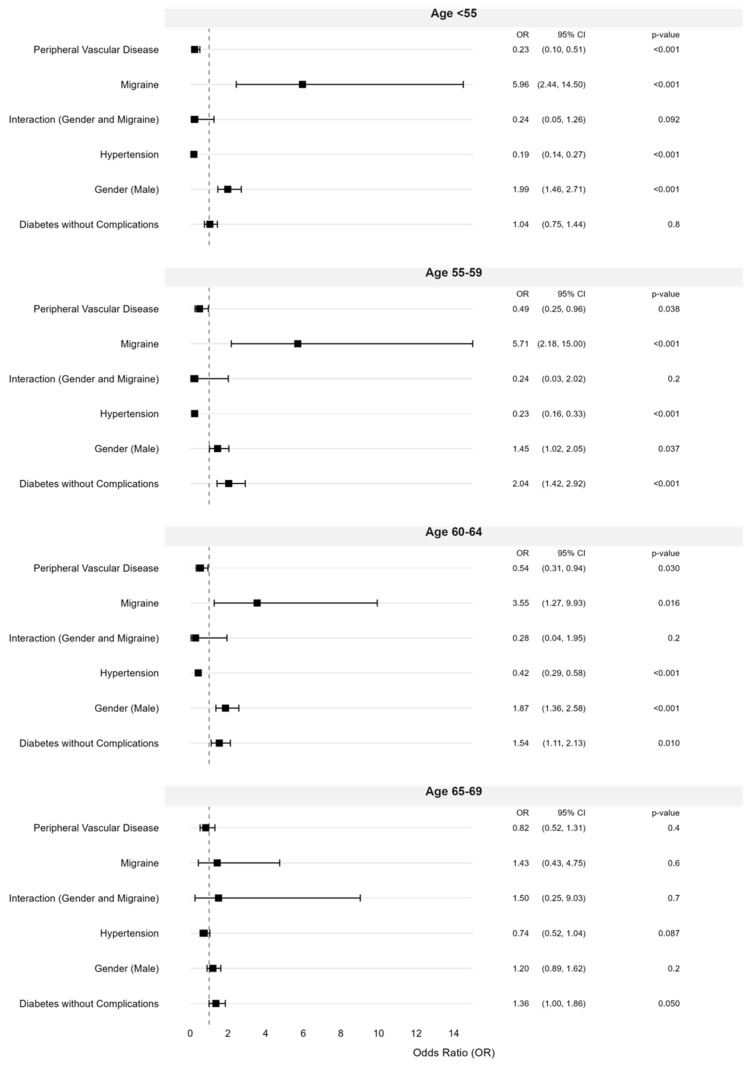
Multivariable Analysis of Odds Ratio by Age Group Compared to >70 Years. This forest plot shows the adjusted Odds Ratio (OR) with 95% confidence intervals (CI) and *p*-values from a multivariable analysis of factors associated with Non-Arteritic Anterior Ischemic Optic Neuropathy (NAION). Age groups (<55, 55–59, 60–64, 65–69) are compared to the reference group (>70 years). Variables include sex (male), interaction between sex and migraine status, peripheral vascular disease, migraine, hypertension, and diabetes without complications. The vertical dashed line marks odds ratio = 1, the reference value representing no association between the examined factor and NAION risk. Squares represent adjusted RR, horizontal lines indicate 95% CI, and statistical significance is noted by *p*-values in the right panel.

Using known predictors for NAION and migraine, we have built sequential multivariable models for the predictors of NAION occurring below age 70 (Figure 3). The strongest association was observed in patients <55 years (OR = 5.96, 95% CI 2.44–14.50, *p* < 0.001), followed by those aged 55–59 (OR = 5.71, 95% CI 2.18–15.00, *p* = 0.001), and 60–64 (OR = 3.55, 95% CI 1.27–9.93, *p* = 0.016). The association was not significant for patients aged 65–69 years (OR = 1.43, 95% CI 0.43–4.75, *p* = 0.6). Although the number of NAION events among migraine patients in the youngest age stratum (<55 years) was limited, the direction and magnitude of the association were consistent across adjacent age groups and remained evident in multivariable and propensity-matched validation analyses. These findings suggest a robust age-dependent pattern rather than reliance on a single subgroup estimate, although effect size estimates in the youngest patients should be interpreted with reduced precision.

### 3.4. Validation of the Age Directionality Outcome Findings by a Propensity Score Model

**Figure 4:**
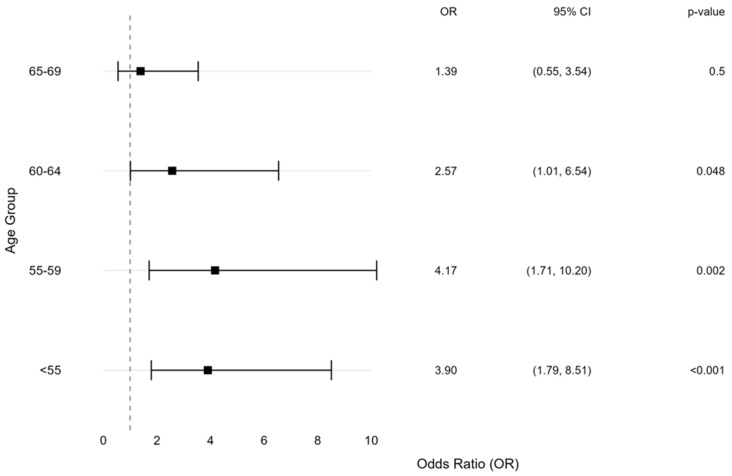
Univariable Analysis of Migraine as a Predictor for Younger Age at NAION Diagnosis in a Matched Cohort. This forest plot presents the Odds Ratio (OR) with 95% confidence intervals (CI) and *p*-values for migraine as a predictor of younger age at Non-Arteritic Anterior Ischemic Optic Neuropathy (NAION) diagnosis. The analysis is based on a matched cohort, with age groups (<55, 55–59, 60–64, 65–69) compared to the reference group (>70 years). The vertical dashed line marks odds ratio = 1, the reference value representing no association between the examined factor and NAION risk. Squares represent OR, horizontal lines indicate 95% CI, and significance is noted by *p*-values.

To validate the association between migraine and young age among female patients, but not among male patients, we conducted a 1:4 propensity-score matched analysis. This analysis matched 62 NAION patients with a history of migraine to 248 NAION patients without migraine (Figure S1, Table S5). After propensity-score matching, the standardized mean differences were less than 0.1 for all variables but younger age at NAION diagnosis for the migraine patients (mean age 62 ± 12 years vs. 68 ± 13, *p* < 0.001). The age at NAION diagnosis was younger among female patients with migraine (mean age 60.4 ± 12 vs. 68.7 ± 12.2 years, *p* < 0.001), whereas no significant difference was observed among male patients (mean age 64.5 ± 12.7 vs. 67 ± 13.6 years, *p* = 0.46). In the matched cohort analysis, migraine was associated with NAION diagnosis in younger patients but not patients aged ≥70 years (Figure 4). The strongest association was observed in patients age 55–59 (OR = 4.17, 95% CI 1.71–10.20, *p* = 0.002), followed by ages <55 (OR = 3.90, 95% CI 1.79–8.51, *p* < 0.001) and 60–64 (OR = 2.57, 95% CI 1.01–6.54, *p* = 0.048), while no significant association was found in the 65–69 age group (OR = 1.39, 95% CI 0.55–3.54, *p* = 0.5).

## 4. Discussion

This case–control study investigated the relationship between migraine and NAION at a national level. While the association between migraine, particularly with aura, and ischemic cerebrovascular events is well-established [22,23,24], the association between migraine and NAION has been less thoroughly explored.

Several findings in this study are directly supported by the data. Migraine was not more prevalent among NAION patients than among matched controls. However, within the NAION cohort, patients with migraine—particularly women—developed NAION at a younger age and exhibited fewer traditional vascular comorbidities. These associations remained consistent across multivariable and propensity-matched analyses, supporting the robustness of the observed age- and sex-specific patterns.

Although not directly tested in the present study, several hypotheses may help contextualize these observations. Migraine has been associated with impaired retinal and choroidal blood flow, altered microvascular density, and dysregulated autoregulation, which could lower the ischemic threshold of the optic nerve head. In this context, vasoconstrictive migraine treatments such as triptans [25,26] or caffeine-containing medications [27] may further reduce perfusion and contribute to ischemic vulnerability.

To assess the independent effect of migraine on NAION risk, we matched cases and controls for established risk factors. Migraine prevalence was slightly higher in the NAION cohort, particularly among younger women, but not significantly so. Notably, among migraine patients, those who developed NAION had a higher prevalence of congestive heart failure (CHF) compared with migraine patients without NAION, despite overall CHF rates being balanced between cohorts. This suggests that CHF-related hypoperfusion may contribute to NAION risk, and that impaired vascular autoregulation in migraine could amplify optic nerve vulnerability to ischemia [5].

Within the NAION cohort, patients with migraine developed NAION at a younger age and had lower rates of diabetes mellitus and PVD, suggesting a potentially distinct underlying pathophysiology in this subgroup. Sex-stratified analyses further demonstrated that women with migraine developed NAION nearly a decade earlier than women without migraine, whereas no such age difference was observed among men. Our findings suggest that migraine may not affect the entire population uniformly but could play a role in specific subpopulations, particularly females <65 years of age.

It appears that migraine by itself may not be an independent risk factor for NAION; however, it might accelerate the onset of NAION in individuals predisposed to the condition, for example, those with structural predispositions due to crowded discs or limited blood flow to the posterior ciliary arteries [2,4]. A prior population study found higher rates of cerebral small vessel disease in NAION patients compared to controls matched for age, sex, and vascular risk factor [28]. This suggests that NAION may be independently associated with cerebral small vessel disease, similar to what the stronger association observed with migraine [22].

Several possible mechanisms may support the hypothesis that migraine serves as a risk factor for NAION. Patients with migraine exhibit reduced retinal and choroidal blood flow as well as vascular density both during and between attacks [29,30,31,32]. Furthermore, impaired autoregulation of the neuro microvasculature has been established in migraine patients [12,13], potentially affecting the posterior ciliary arteries adversely. Another contributing factor is the impaired autoregulation of larger cerebral blood vessels. Rapid dilation of carotid branches may result in compression of smaller, less compliant vessels [12,13,22,23,24]. Moreover, vasoconstriction from migraine treatments like triptans [25,26] or caffeine-containing drugs [27] could additionally reduce optic nerve head blood flow, potentially triggering NAION. A previous study found that a prominent constrictive effect of triptans on the extracranial cerebral vasculature, and especially on the middle meningeal artery [33]. Consequently, the flow through the ophthalmic artery may be reduced whenever the ophthalmic artery either arises from or receives collateral flow [34] from the middle meningeal artery [35].

Ocular blood flow is autoregulated to ensure consistent delivery of oxygen and nutrients across varying hemodynamic conditions [18]. Several estrogen derivatives enhance ocular blood flow by maintaining improved autoregulation in both the cerebrovascular and ophthalmic vascular systems, leading to increased blood flow in the retinal, retrobulbar, and choroidal vessels [36,37]. This hormonal influence has been demonstrated using laser Doppler flowmetry [38]. Young premenopausal women (<40 years) were shown to have higher choroidal sub-macular blood flow compared to women over 40, but such age-related difference was not observed in men [36]. Our findings indicate that female migraine patients were more likely to experience NAION at a younger age. The lack of this age-related pattern in men suggests that migraine alone may not directly cause NAION, pointing instead to a complex interaction between hormonal and vascular mechanisms. We observed that the strongest association between migraine and NAION occurs during perimenopause and early post-menopause (ages 50–60), which is consistent with the typical onset of menopause (ages 50–54) [34]. Additionally, the gradual weakening of this association as women approach the typical age for NAION onset further supports the hypothesis of hormonal involvement in the relationship between migraine and NAION.

We therefore hypothesize that migraine-associated vascular dysregulation may lower the ischemic threshold of the optic nerve head. While in women estrogen’s protective effects seem to counterbalance these vascular vulnerabilities, the decline in estrogen levels during menopause may reveal these underlying susceptibilities, potentially triggering ischemic events such as NAION during this period. This mechanism aligns with the heightened risk of cerebrovascular events observed in early menopause [39].

There are several limitations to our study. The retrospective design and reliance on ICD-9 codes may introduce misclassification bias and data inconsistencies. Additionally, our data does not encompass neuro-ophthalmological findings, such as the cup-to-disc ratio or the presence of drusen, which may influence the occurrence of NAION [40]. Moreover, although CGA as a cause of arteritic AION was not present in any of the patients in our cohort, it cannot be ruled out as a possible misdiagnosis of NAION. However, it is a rare entity and could have affected a minority of cases in both migraineurs and non-migraineurs alike. Although this study represents one of the largest national NAION cohorts reported, the subgroup of patients with migraine was relatively small, indicating potential underdiagnosis of migraine and reducing statistical power. Nonetheless, while migraine may be underdiagnosed in individuals from lower socioeconomic backgrounds [41], the diagnosis of NAION in our study was not influenced by socioeconomic status. Given the hypothesis-driven nature of the age-stratified analyses and the consistency of findings across complementary modeling approaches, formal correction for multiple comparisons was not applied. Nevertheless, results derived from smaller subgroups should be interpreted cautiously, with emphasis placed on the reproducibility of age-related trends rather than on individual point estimates. Importantly, nondifferential misclassification of migraine or NAION would be expected to bias associations toward the null rather than generate spurious age-specific effects. Additionally, the lack of comprehensive data on the use of hormone replacement therapy prevented us from fully elucidating the specific roles of the menstrual cycle as well as menopause and its medical management in pathophysiology. The potential impacts of female sex and migraine on our findings were also considered, as these factors could introduce bias. To address this, we employed two complementary approaches: adding an interaction variable to the regression models and conducting validation using propensity score matching to ensure robustness in the interpretation of findings. Information on migraine subtype, attack frequency, and acute or preventive migraine medications was unavailable, limiting phenotype-specific inference.

## 5. Conclusions

This population-based case–control study found no direct association between migraine and NAION risk; however, NAION patients with migraine had fewer cardiovascular comorbidities, suggesting a possible association with migraine. Sub-analysis found that diagnosis of NAION occurred significantly earlier in female patients with a history of migraine than in women without this history, and that females with migraine had fewer traditional NAION risk factors. These findings support potential sex-specific mechanisms, possibly involving the interaction between migraine-related vascular dysregulation and hormonal factors. Future studies integrating migraine phenotype and medications, hormonal status, and direct measures of ocular and cerebral perfusion are required to directly test these mechanistic hypotheses.

## Figures and Tables

**Table 1:** Baseline characteristics of migraine patients with and without NAION.

Characteristic	NAION N = 62	W/O NAION, N = 212	*p*-Value
Age mean (SD)	62 (12)	60 (11)	0.24
Female sex	45 (72.6%)	144 (68%)	0.59
Socioeconomic Status			0.09
High	18 (29%)	41 (19%)	
Medium	36 (58%)	120 (57%)	
Low	4 (6.5%)	39 (18%)	
No data	4 (6.5%)	12 (5.7%)	
Hypertension	35 (56.4%)	91 (43%)	0.083
Any DM	22 (35.5%)	94 (44.6%)	0.27
DM without Complications	17 (27.4%)	80 (38%)	0.18
DM with Complications	5 (8%)	14 (6.6%)	0.9
Chronic Pulmonary Disease	19 (30.6%)	69 (33%)	0.9
Cerebrovascular Disease	14 (22.6%)	50 (18%)	0.88
Rheumatic Disease	4 (6.4%)	17 (8%)	0.89
History of Malignancy	8 (12.9%)	19 (9%)	0.5
Any Liver Disease	13 (21%)	31 (14.1%)	0.32
Mild Liver Disease	12 (19.3%)	29 (13.7%)	0.37
Moderate/severe Liver Disease	1 (1.6%)	2 (1%)	1
Congestive Heart Failure	6 (9.7%)	5 (2.4%)	0.027
Dementia	3 (4.8%)	12 (5.7%)	1.0
Peripheral Vascular Disease	1 (1.6%)	14 (6.6%)	0.23

**Table 2:** Baseline characteristics of NAION patients with and without migraine stratified by sex.

	Female, N = 739			Male, N = 890		
Characteristic	W/O Migraine, N = 694 ^1^	Migraine, N = 45 ^1^	*p*-Value ^2^	W/O Migraine, N = 873 ^1^	Migraine, N = 17 ^1^	*p*-Value ^2^
Socioeconomic Status			0.17			0.11
High	139 (20%)	14 (31%)		155 (18%)	4 (24%)	
Medium	388 (56%)	25 (56%)		554 (63%)	11 (65%)	
Low	130 (19%)	4 (8.9%)		128 (15%)	0 (0%)	
Age at NAION event	69 (12)	60 (12)	<0.001	65 (13)	65 (13)	0.58
Ethnicity (Jewish)	555 (80%)	39 (87%)	0.27	741 (85%)	16 (94%)	0.49
Hypertension	449 (65%)	25 (56%)	0.21	544 (62%)	10 (59%)	0.76
Any DM	387 (56%)	16 (35%)	0.013	504 (58%)	6 (35%)	0.1
DM without Complications	301 (43%)	12 (27%)	0.028	390 (45%)	5 (29%)	0.21
DM with Complications	86 (12%)	4 (8.9%)	0.48	114 (13%)	1 (5.9%)	0.71
Chronic Pulmonary Disease	203 (29%)	17 (38%)	0.22	209 (24%)	2 (12%)	0.38
Cerebrovascular Disease	144 (21%)	11 (24%)	0.55	206 (24%)	3 (18%)	0.77
Rheumatic Disease	102 (15%)	3 (6.7%)	0.13	56 (6.4%)	1 (5.9%)	1.00
Malignancy	75 (11%)	6 (13%)	0.62	107 (12%)	2 (12%)	1.00
Mild Liver Disease	74 (11%)	9 (20%)	0.05	75 (8.6%)	3 (18%)	0.18
Congestive Heart Failure	61 (8.8%)	3 (6.7%)	0.78	103 (12%)	3 (18%)	0.44
Dementia	48 (6.9%)	2 (4.4%)	0.76	38 (4.4%)	1 (5.9%)	0.53
Peripheral Vascular Disease	44 (6.3%)	0 (0%)	0.10	107 (12%)	1 (5.9%)	0.71
Moderate/Severe Liver Disease	3 (0.4%)	1 (2.2%)	0.22	2 (0.2%)	0 (0%)	1.00

^1^ Mean (SD); n (%). ^2^ *T*-test; Fisher’s exact test; Pearson’s Chi-squared test.

## Data Availability

The data presented in this study are available on request from the corresponding author. The data are not publicly available due to patient confidentiality and institutional restrictions.

## Supplementary Materials

The following supporting information can be downloaded at: https://www.mdpi.com/article/10.3390/brainsci16010082/s1.
