# Supplementary material for: Is Migraine a Risk Factor for Non-Arteritic Anterior Ischemic Optic Neuropathy? Insights from a National Case–Control Study
Source: Brain Sci. 2026 Jan 7;16(1):82. doi: 10.3390/brainsci16010082 (PMC12838819; doi:10.3390/brainsci16010082)
Supplement: Supplementary file 1 [file brainsci-16-00082-s001.zip › brainsci-4013291-supplementary.pdf]

Supplementary material

Appendix

Supplementary Table 1. List of ICD-9 Codes

**Characteristic**		**ICD-9 Diagnosis/ Procedure/ ATC-5 Code**
**Hypoperfusion states**		
Reduced systemic vascular resistance	Sepsis	995.9x
	Septic shock	782.52
	Pyelonephritis	590.x
	Peritonitis	567.x
	Septic arthritis	711.0x, 711.9x
	Necrotizing fasciitis	728.86
	Pneumonia	481.x, 482.x, 483.x, 484.x, 486.x, 507.x, 510.x, 511.x, 513.x
	Cholecystitis	574.0x, 574.1x, 574.3x, 574.4x, 574.6x, 574.7x, 574.8x, 575.0, 575.1x, 575.2
	Cholangitis	576.1
	Anaphylactic shock	995.0
	Vasopressor support	C05AX06, C01CA07, C01CA24, C01CA04, C01CA03, H01BA01, H01BA02
Cardiac dysfunction	Cardiac arrest	427.5, Z9960
	Myocardial infarction	410.x, 411.x, 412.x, Z88.57
	Acute pulmonary edema	518.4
	Pulmonary embolism	415.1x, 673.x
	Cardiogenic shock	785.51
Hypovolemia	GI bleeding	578.x, 456.0, Z44.4x
	Trauma	860.x-869.x, 959.0x, 959.1x, 959.2, 959.3, 959.6, 959.7, 959.8, 959.9
	Hypotension	458.x
	Hemodialysis	Z39.95

	Major surgery	CABG: Z36.1x<br>ECMO: Z39.6x<br>Valvuloplasty: Z39.6x<br>Colectomy: Z35.1x, Z35.2x<br>Gastrectomy: Z43.5, Z43.6, Z43.7, Z43.8x, Z43.9x, Z44.3x<br>Cholecystectomy: Z51.2x<br>ERCP: Z51.10<br>Spine surgeries: Z81.3x<br>Femur surgery: Z79.05, Z79.15, Z79.25, Z79.35, Z79.45, Z79.55, Z79.65, Z79.95
	Pneumothorax	512.x, 860.x
Others	Syncope	780.2
	Aortic dissection	441.x
	Shock	785.5x, 958.4x, 995.0, 995.4, 995.6x, 999.4x, 999.5x
**Systemic comorbidities**		
Liver disease		571.2, 571.4-571.6, 456.0-456.21, 572.2-572.8
Diabetes mellitus		250.x
Renal disease		403.01, 403.11, 403.91, 404.02, 404.03, 404.12, 404.13, 404.92, 404.93, 582.x, 583.0-583.7, 585.x, 586.x, 588.0
Malignancy		140.x-172.x, 174.x-195.8, 200.x-208.x, 238.6, 196.x-199.x
Chronic pulmonary disease		416.8, 416.9, 490.x-505.x, 506.4, 508.1, 508.8
Myocardial infarction		410.x, 411.x, 412.x, Z88.57
Congestive heart failure		398.91, 402.01, 402.11, 402.91, 404.01, 404.03, 404.11, 404.13, 404.91, 404.93, 425.4-425.9, 428.x
Dementia		290.x, 294.1, 331.2
Non-arteritic anterior ischemic optic neuropathy		377.41
Cerebrovascular disease		430.x-438.x, 362.34

GI, gastrointestinal; CABG, coronary artery bypass graft; ECMO, extracorporeal membrane oxygenation; ERCP, endoscopic retrograde cholangiopancreatography

Supplementary Table 2. Baseline characteristics of NAION patients (cases) and matched controls

**Variable**	**Control**, N = 6,433[1]	**NAION** = 1,629[1]	**Difference**[2]	**p-value**[3]
**Sex (Female)**	2,911 (45%)	739 (45%)	0.00	0.93
**Socioeconomic Status**			0.04	
High	1,136 (18%)	312 (19%)		0.453
Medium	3,883 (60%)	978 (60%)		0.237
Low	1,102 (17%)	262 (16%)		0.124
**Age at NAION event**	67 (13)	67 (13)	0.04	0.109
**Hypertension**	3,456 (54%)	1,028 (63%)	0.19	**<0.001**
**Diabetes without Complications**	2,789 (43%)	708 (43%)	0.00	0.93
**Diabetes with Complications**	733 (11%)	205 (13%)	0.04	0.181
**Chronic Pulmonary Disease**	1,692 (26%)	431 (26%)	0.00	0.98
**Cerebrovascular Disease**	1,371 (21%)	364 (22%)	0.03	0.36
**Malignancy**	708 (11%)	190 (12%)	0.02	0.45
**Congestive Heart Failure**	602 (9.4%)	170 (10%)	0.04	0.18
**Mild Liver Disease**	558 (8.7%)	161 (9.9%)	0.04	0.13
**Peripheral Vascular Disease**	531 (8.3%)	152 (9.3%)	0.04	0.16
**Migraine**	212 (3.3%)	62 (3.8%)	0.03	0.3
**Moderate/Severe Liver Disease**	42 (0.7%)	6 (0.4%)	0.04	0.2
**HIV**	3 (<0.1%)	0 (0%)	0.03	0.99

[1]Mean (SD); n (%)

[2]Standardized Mean Difference

[3] Mixed logistic model

Supplementary Table 3. Multivariable Logistic Regression Analysis of Risk Factors for Non-Arteritic Anterior Ischemic Optic Neuropathy (NAION)

**Characteristic**	**OR**[1]	**95% CI**[1]	**p-value**
Hypertension	1.69	1.47, 1.93	**<0.001**
Migraine	1.17	0.87, 1.57	0.3
Congestive Heart Failure	1.37	0.97, 1.92	0.071
Diabetes without Complications	0.56	0.30, 1.05	0.070
Peripheral Vascular Disease	1.62	1.09, 2.41	**0.018**

[1]OR = Odds Ratio, CI = Confidence Interval

Supplementary Table 4. Baseline characteristics of Control cohort patients with and without migraine

Variable	W/O migraine N = 6,221[1]	Migraine patients, N = 212[1]	Difference[2]	p-value[3]
**Birth Year**	1,945 (14)	1,956 (12)	-0.79	**<0.001**
**Age (SD)**	68 (13)	60 (11)	0.58	**<0.001**
**Male sex**	3,454 (56%)	68 (32%)	0.49	**<0.001**
**Socioeconomic Status**			0.08	0.7
High	1,095 (18%)	41 (19%)		
Low	1,063 (17%)	39 (18%)		
Medium	3,763 (60%)	120 (57%)		
**Ethnicity**			0.2	**0.002**
Arab	734 (12%)	40 (19%)		
Jewish	5,487 (88%)	172 (81%)		
**Hypertension**	3,365 (54%)	91 (43%)	0.22	**<0.001**
**Congestive Heart Failure**	597 (9.6%)	5 (2.4%)	0.31	**<0.001**
**HIV**	3 (<0.1%)	0 (0%)	0.03	>0.9
**Dementia**	434 (7.0%)	12 (5.7%)	0.05	0.5
**Mild Liver Disease**	529 (8.5%)	29 (14%)	0.17	**0.008**
**Moderate/Severe Liver Disease**	40 (0.6%)	2 (0.9%)	0.03	0.6
**Peripheral Vascular Disease**	517 (8.3%)	14 (6.6%)	0.07	0.4
**Rheumatic Disease**	275 (4.4%)	17 (8.0%)	0.15	**0.013**
**Any Diabetes**	3428 (55%)	94 (44.3%)	0.14	**0.002**
**Diabetes with Complications**	719 (12%)	14 (6.6%)	0.17	**0.026**
**Diabetes without Complications**	2,709 (44%)	80 (38%)	0.12	0.093
**Malignancy**	689 (11%)	19 (9.0%)	0.07	0.3
**Chronic Pulmonary Disease**	1,623 (26%)	69 (33%)	0.14	**0.036**
**Cerebrovascular Disease**	1,321 (21%)	50 (24%)	0.06	0.4

[1] Mean (SD); n (%) [2] Standardized Mean Difference [3] Wilcoxon rank sum test; Pearson's Chi-squared test; Fisher's exact test

S**upplementary Table 5.** Matched population, baseline covariates among NAION patients with history of migraines prior to the NAION event and those without

**Variable**	No, N = 248[1]	Yes, N = 62[1]	**SMD**[2]	**95% CI**[23]	**p-value**[4]
**Sex (**Female)	180 (73%)	45 (73%)	0.00	-0.28, 0.28	>0.9
**Socioeconomic Status**			0.00	-0.28, 0.28	>0.9
High	72 (29%)	18 (29%)			
Low	16 (6.5%)	4 (6.5%)			
Medium	144 (58%)	36 (58%)			
**Age at NAION diagnosis**	68 (13)	62 (12)	0.54	0.26, 0.82	**<0.001**
**Ethnicity (**Jewish)	220 (89%)	55 (89%)	0.00	-0.28, 0.28	>0.9
**Hypertension**	140 (56%)	35 (56%)	0.00	-0.28, 0.28	>0.9
**Congestive Heart Failure**	23 (9.3%)	6 (9.7%)	0.01	-0.26, 0.29	>0.9
**Dementia**	19 (7.7%)	3 (4.8%)	0.12	-0.16, 0.40	0.6
**Mild Liver Disease**	20 (8.1%)	12 (19%)	0.33	0.05, 0.61	**0.009**
**Moderate/Severe Liver Disease**	1 (0.4%)	1 (1.6%)	0.12	-0.16, 0.40	0.4
**Peripheral Vascular Disease**	4 (1.6%)	1 (1.6%)	0.00	-0.28, 0.28	>0.9
**Rheumatic Disease**	35 (14%)	4 (6.5%)	0.25	-0.02, 0.53	0.10
**Diabetes with Complications**	20 (8.1%)	5 (8.1%)	0.00	-0.28, 0.28	>0.9
**Diabetes without Complications**	94 (38%)	17 (27%)	0.22	-0.05, 0.50	0.12
**Malignancy**	29 (12%)	8 (13%)	0.04	-0.24, 0.32	0.8
**Chronic Pulmonary Disease**	72 (29%)	19 (31%)	0.04	-0.24, 0.31	0.8
**Cerebrovascular Disease**	53 (21%)	14 (23%)	0.03	-0.25, 0.31	0.8
**Obstructive Sleep Apnea**	1 (0.4%)	1 (1.6%)	-0.12	-0.40,0.16	0.4

[1]Mean (SD); n (%)

[2]Standardized Mean Difference

[3]CI = Confidence Interval

[4] t-test; Pearson's Chi-squared test; Fisher's exact test

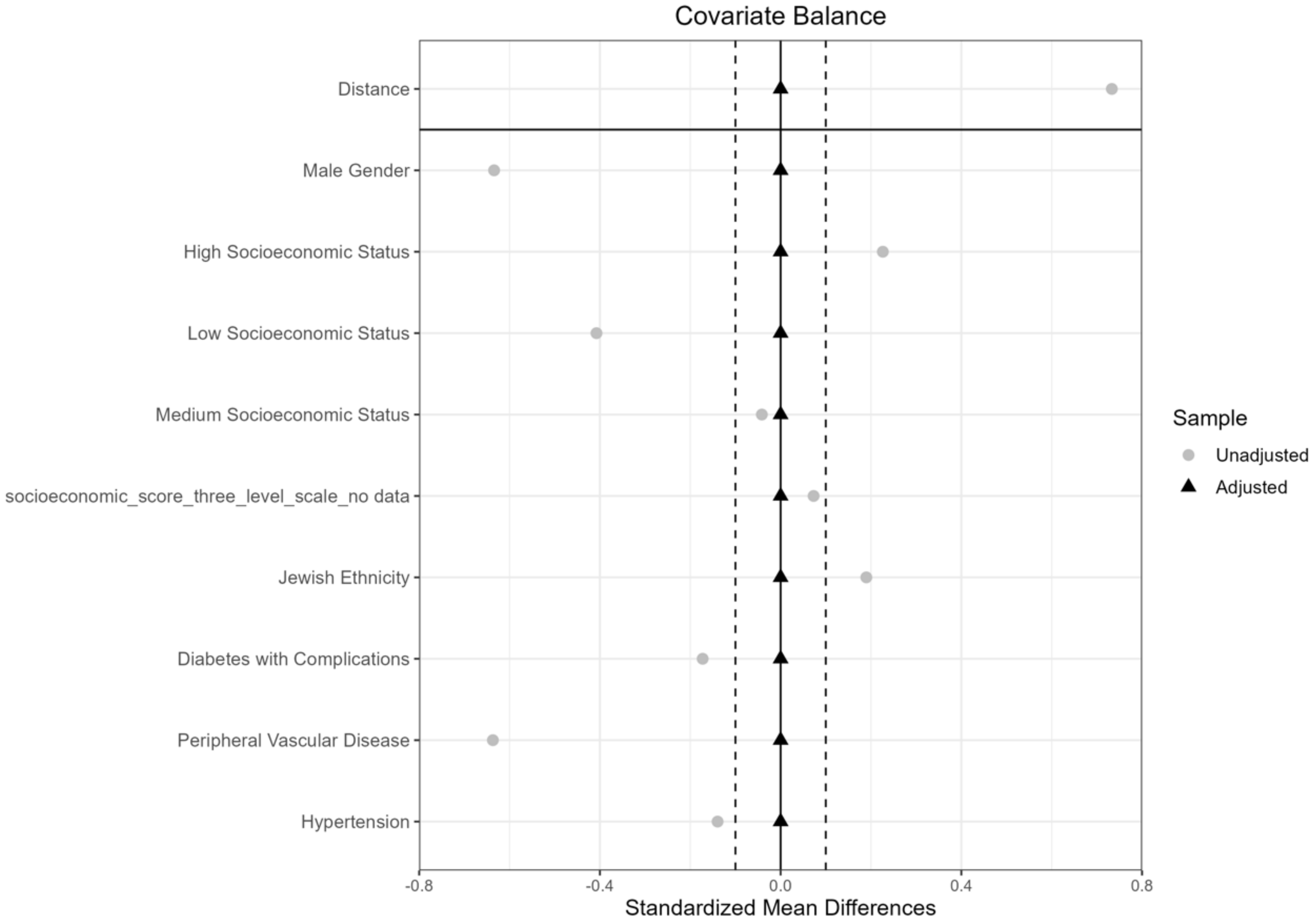

Supplementary Figure 1. Covariate Balance Before and After Propensity Score Matching

This Love plot illustrates the standardized mean differences (SMD) of covariates before (unadjusted, gray circles) and after (adjusted, black triangles) propensity score matching. The vertical dashed lines at ±0.1 represent the threshold for acceptable balance. Covariates included are distance, sex, socioeconomic status (low, medium, high, and missing data), Jewish ethnicity, diabetes with complications, peripheral vascular disease, and hypertension.
